# Aberrant Autophagic Response in The Muscle of A Knock-in Mouse Model of Spinal and Bulbar Muscular Atrophy

**DOI:** 10.1038/srep15174

**Published:** 2015-10-22

**Authors:** Paola Rusmini, Maria Josefa Polanco, Riccardo Cristofani, Maria Elena Cicardi, Marco Meroni, Mariarita Galbiati, Margherita Piccolella, Elio Messi, Elisa Giorgetti, Andrew P. Lieberman, Carmelo Milioto, Anna Rocchi, Tanya Aggarwal, Maria Pennuto, Valeria Crippa, Angelo Poletti

**Affiliations:** 1 Dipartimento di Scienze Farmacologiche e Biomolecolari (DiSFeB), Centro di Eccellenza sulle Malattie Neurodegenerative, Università degli Studi di Milano, Milano, Italy; 2 Dulbecco Telethon Institute, Centre for Integrative Biology (CIBIO), Università degli Studi di Trento, Trento, Italy; 3 Department of Pathology, University of Michigan, Ann Arbor, Michigan, USA; 4 Neuroscience and Brain Technologies Department, Istituto Italiano di Tecnologia, Genova, Italy; 5 Dipartimento di Medicina Sperimentale, Università degli Studi di Genova, Genova; 6 Laboratory of Experimental Neurobiology, C. Mondino National Neurological Institute, Pavia, Italy; 7 Centro InterUniversitario sulle Malattie Neurodegenerative, Università degli Studi di Firenze, Genova, Roma Tor Vergata and Milano, Italy

## Abstract

Spinal and bulbar muscular atrophy (SBMA) is characterized by loss of motoneurons and sensory neurons, accompanied by atrophy of muscle cells. SBMA is due to an androgen receptor containing a polyglutamine tract (ARpolyQ) that misfolds and aggregates, thereby perturbing the protein quality control (PQC) system. Using SBMA AR113Q mice we analyzed proteotoxic stress-induced alterations of HSPB8-mediated PQC machinery promoting clearance of misfolded proteins by autophagy. In muscle of symptomatic AR113Q male mice, we found expression upregulation of Pax-7, myogenin, E2-ubiquitin ligase UBE2Q1 and acetylcholine receptor (AchR), but not of MyoD, and of two E3-ligases (MuRF-1 and Cullin3). TGFβ1 and PGC-1*α* were also robustly upregulated. We also found a dramatic perturbation of the autophagic response, with upregulation of most autophagic markers (Beclin-1, ATG10, p62/SQSTM1, LC3) and of the HSPB8-mediated PQC response. Both HSPB8 and its co-chaperone BAG3 were robustly upregulated together with other specific HSPB8 interactors (HSPB2 and HSPB3). Notably, the BAG3:BAG1 ratio increased in muscle suggesting preferential misfolded proteins routing to autophagy rather than to proteasome. Thus, mutant ARpolyQ induces a potent autophagic response in muscle cells. Alteration in HSPB8-based PQC machinery may represent muscle-specific biomarkers useful to assess SBMA progression in mice and patients in response to pharmacological treatments.

Spinal and bulbar muscular atrophy (SBMA), or Kennedy’s disease, is an inherited X-linked motoneuron disease (MND) characterized by motoneurons loss in the anterior horns of the spinal cord and in the brain stem, together with atrophy of bulbar, facial and limb muscles^1^,^2^,^3^. Dorsal root ganglia sensory neurons are also affected resulting in sensory function alterations^4^,^5^,^6^,^7^. Despite being classified as MND, there is evidence that also muscle cells are directly affected in SBMA, suggesting that SBMA might be considered a neuromuscular disease rather than a MND^8^,^9^,^10^,^11^,^12^,^13^,^14^,^15^,^16^,^17^,^18^.

SBMA has been linked to the expansion of a CAG triplet repeat sequence located in the exon 1 of the androgen receptor (AR) gene^19^. The CAG tandem repeat codes for a polyglutamine (polyQ) tract in the N-terminus of the AR protein. In the normal population, the polyQ tract ranges from 9 to 37 Qs (average = 22), while in SBMA patients the polyQ tract is longer than 38 Qs, up to 68 contiguous Qs (ARpolyQ)^20^,^21^. Although the function of the polyQ tract is still unknown, it has been proposed that the polyQ tract may serve as a transcriptional regulatory domain^22^,^23^,^24^. PolyQ expansions have been described in eight other totally unrelated proteins involved in neurodegenerative diseases (CAG/polyQ diseases)^25^, suggesting that the polyQ expansion confers a common gain of neurotoxic function(s) to the mutant proteins. Toxicity has been linked to the acquisition of polyQ-induced aberrant protein conformation (misfolding), which leads to protein aggregation. This process may perturb the protein quality control (PQC) system in cells expressing high levels of ARpolyQ. Bulbar and spinal cord motoneurons, dorsal root ganglia neurons and skeletal muscle cells are all postmitotic cells^26^,^27^, and express high levels of AR^28^, which may make them particularly sensitive to the accumulation of ARpolyQ misfolded species. The nature of the selective cell vulnerability in ARpolyQ and the precise motoneuronal *versus* muscular contribution to disease is still unclear.

There is emerging evidence supporting the concept that muscle is a key component of disease pathogenesis. Expression of a muscle-specific insulin-like growth factor 1 (IGF-1) isoform selectively in muscle attenuated disease progression in SBMA mice^29^. More recently, using a neurogenic or myogenic tet-On and Cre-loxP based approach to express ARpolyQ in either motor neurons (NeuroAR) or myocytes (MyoAR) in transgenic mice, Monks and coll. have shown motor function defects in NeuroAR mice, but not in MyoAR mice. Expression of ARpolyQ in muscle of MyoAR mice resulted in a more pronounced reduction in the size of fast glycolytic fibers. The expression of muscle BDNF was reduced in androgen-dependent manner in both models, paralleling changes in motor function, thus supporting the notion that growth factors alteration may play a role in muscle in response to ARpolyQ direct or indirect toxicity^18^. Nonetheless, the overexpression of human wild type AR (wtAR) in the skeletal muscle of wt mice is sufficient to recapitulate several aspects of SBMA^11^,^13^,^30^,^31^. Moreover, muscle-specific silencing of the mutant ARpolyQ expression in different SBMA mouse models resulted in prolonged survival, thereby proving evidence for a direct effect of ARpolyQ on muscle atrophy^32^,^33^.

Muscle is a direct target of the anabolic androgenic action mediated by the AR^34^,^35^. ARpolyQ toxicity strictly depends on androgens, possibly because testosterone triggers the switch of ARpolyQ structure from a “non-toxic” to a “toxic” conformation. Aberrant conformational modifications may generate ARpolyQ aggregates, that are not necessarily toxic *per se*^36^. Testosterone binding induces nuclear translocation of misfolded ARpolyQ, and nucleus is the cellular compartment where ARpolyQ exerts most of its toxic effects^37^,^38^. Interestingly, in adipose mesenchymal cells derived from SBMA patients, inhibition of the proteasome, but not of autophagy, resulted in ARpolyQ accumulation into nuclear aggregates^39^. However, the ARpolyQ protein reduces long-term protein turnover and blocks the cytoplasmic autophagic flux^40^,^41^,^42^,^43^,^44^,^45^,^46^. Pharmacological restoration of a normal autophagic flux, obtained by the transcription factor EB (TFEB) activator trehalose^47^, greatly increased the clearance of mutant misfolded ARpolyQ^43^,^44^. Autophagy flux blockage has also been reported in SBMA mice^40^, and the importance of autophagy in SBMA is sustained by several studies^40^,^48^. However, the response to ARpolyQ toxicity in SBMA muscle is complex and rather controversial. Autophagy dysregulation occurs in muscle of AR113Q knock-in SBMA mice already in basal conditions, but autophagy dysregulation is exacerbated in starved mice or in those undergoing to extensive physical exercise; these dysregulation include peculiar transcriptional modulation of autophagic genes induced by the physiological antagonists TFEB and ZKSCAN3^10^. Among the genes regulated by TFEB and increased in SBMA mice in basal conditions is that coding for TFEB itself. Notably, TFEB-target genes, including LC3, Vps11, Vps18 and Lamp1, are also upregulated in muscle samples derived from SBMA patients^10^. Notably, inhibition of Beclin-1-mediated autophagy activation in AR113Q knock-in SBMA mice diminished skeletal muscle atrophy, extending lifespan and ameliorating their phenotype. Conversely, over-activation of autophagy worsened phenotype^9^. Thus, restoration of normal flux and normalization of dysregulated autophagy are both essential for ARpolyQ clearance in the skeletal muscle of SBMA mice.

Pathways that re-route ARpolyQ to degradation mediated by ubiquitin-proteasome system (UPS) may also be of relevance for its clearance. A delicate equilibrium exists between UPS and autophagy, as both pathways are essential for proper functions of the PQC system^42^,^43^,^46^,^49^. This equilibrium is regulated by a number of proteins, including the co-chaperones BAG1 (for UPS clearance) and BAG3 (for autophagic clearance)^50^. In addition, flux restoration and autophagy modulation can be regulated by few specific chaperones, like some members of the small HSP family (HSPBs), such as HSPB8. Notably, mutations in HSPB8 (at K141 with E or N) have been linked to Charcot-Marie-Tooth type 2L disease and hereditary distal motor neuropathy type II (dHMNII)^51^,^52^. By interacting with BAG3 (in a 2:1 ratio) HSPB8 decreases ARpolyQ aggregation, by increasing its solubility and clearance^43^. HSPB8 also acts on several other misfolded proteins involved in neurodegenerative diseases^43^,^49^,^53^,^54^. HSPB8 acts as facilitator, rather than activator of autophagy^43^. This requires the interaction of 2x(HSPB8)/BAG3 with HSC70/CHIP, allowing misfolded substrate ubiquitination for its p62 mediated recognition and insertion into autophagosomes^53^,^55^. This effect is not associated with mutant dHMNII-HSPB8^56^. In ALS mice, both anterior horn spinal cord motor neurons and the skeletal muscle cells respond to proteotoxicity by activating a robust HSPB8-mediated PQC system response^53^,^57^,^58^. Nothing is known about the involvement of the HSPB8 and BAGs machinery in SBMA skeletal muscle, and the identification of specific autophagy related molecular markers of skeletal muscle degeneration in SBMA might represent a diagnostic valuable tool for monitoring the disease progression. For these reasons, in this study, we have provided an extensive characterization of the autophagic activation as well as of the HSPB8 machinery in the PQC system response in SBMA muscle.

## Results

Here, we investigated the specific response of the HSPB8-mediated PQC machinery to ARpolyQ toxicity in the skeletal muscle of a knock-in mouse model of SBMA. SBMA mice have been obtained by knocking in a segment of the human exon 1 carrying 113 CAG repeats, into the homolog region of the endogenous mouse AR gene (AR113Q mice)^59^. Thus, being controlled by the endogenous AR promoter, these mice retained the physiological levels of AR proteins and AR cell localization in all tissues^59^. For the purpose of our study, this aspect provides a significant advantage over other SBMA mouse models developed using exogenous promoters to drive the unspecific overexpression of exogenous human AR. Over-expression and incorrect cell production of the toxic ARpolyQ might induce unspecific abnormalities in the PQC system response, especially in skeletal muscle tissue, which is particularly sensitive to the presence of altered proteins^58^,^60^.

### Muscle atrophy and altered motor behavior are overt at 24 weeks of age in AR113Q knock-in mice

**Figure 1:**
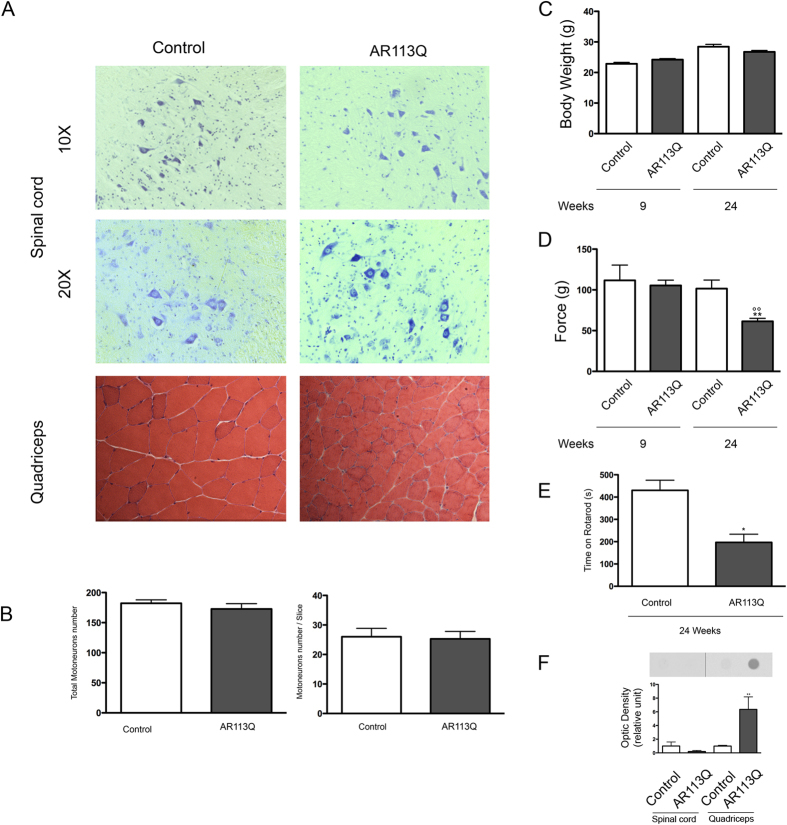
Preliminary characterization of the SBMA mouse model. (**a**) Spinal cord and quadricep cross sections of control and AR113Q male mice at 24 weeks of age (corresponding to symptomatic stage). Nissl staining of lumbar spinal cord shows the cell body of lower motor neurons. Hematoxylin-Eosin staining of quadriceps underscores a marked decrease in fiber size of AR113Q mice compared to control mice. (**b**) Left Panel: average number of total motor neurons in the lumbar spinal cord of control and AR113Q mice at 24 weeks of age (control mice, n = 5; AR113Q, n = 6); Right panel: average number of motor neurons for each slice (number of slices = 7). (**c**) Measurement of body weight in control and AR113Q mice at 9 and 24 weeks of age (control mice, n = 5; AR113Q, n = 20). (**d**) Grip strength test in control and AR113Q mice at 9 and 24 weeks of age (control mice, n = 5; AR113Q, n = 15). **p < 0.001 *vs*. 24 weeks control mice; °°p < 0.001 *vs*. 9 weeks AR113Q mice. (**e**) Rotarod test in control and AR113Q male mice at 24 weeks of age. *p < 0.05 *vs*. 24 weeks control mice (control mice, n = 5; AR113Q, n = 15). (**f**) Filter retardation assay in spinal cord and quadriceps of control and AR113Q male mice at 24 weeks. Androgen receptor AR aggregation was visible only in ARQ113 quadriceps at 24 weeks of age. **p < 0.001 *vs*. 24 weeks quadriceps of control mice.

To evaluate disease manifestations in AR113Q knock-in mice, we analyzed spinal cord and muscle pathology at 24 wks of age. In agreement with previous reports^9^,^10^,^32^,^59^, the histopathological analysis (Fig. 1(a), upper and middle insets) of the anterior horn of the spinal cord excluded the presence of motoneuronal loss in AR113Q male mice at 24 wks of age. This was confirmed by the statistical evaluation of the total number of motoneurons presents at this age (Fig. 1(b)) both in the tissue and in average number for the slice considered (left and right panels, respectively). Conversely, histological analysis of quadriceps muscle (Fig. 1(a), lower insets) revealed signs of myopathy. Hematoxylin and Eosin analysis showed that the quadriceps of AR113Q mice was characterized by the presence of smaller fibers compared to those present in age-matched male control mice. It has been previously showed that the functional deficit on behavioral testing of AR113Q males is also accompanied by morphologic changes in skeletal muscle which are indicative of both neurogenic and myopathic effects in a way that resemble the mixed features described in biopsies of Kennedy’s disease muscle (29, 30). It has been shown that in these mice myopathy occurs before overt spinal cord pathology^59^,^61^. In addition, the body weight of AR113Q mice was decreased compared to control mice (9 wks = controls 22.86 ± 0.4417, AR113Q male mice 24.21 ± 0.342; 24 wks = controls 28.45 ± 0.7571, AR113Q male mice 26.75 ± 0.4581) since SBMA mice tended to grow less than control (Fig. 1(c)). To assess motor behavior in comparison with literature data^9^,^10^,^32^,^59^,^61^, we performed grip strength (Fig. 1(d)) and rotarod (Fig. 1(e)) analyses. We confirmed that AR113Q male mice do not show motor function abnormalities at 9 wks of age. On the other hand, AR113Q mice showed reduced performance at 24 wks of age (mean ± SEM grip strength test: 9 wks = controls 111.7 ± 18.82, AR113Q male mice 105.4 ± 6.566; 24 wks = controls 101.5 ± 10.53, AR113Q male mice 61.42 ± 3.667 p < 0.001 vs control 24wks; p < 0.001 vs AR113Q 9wks. Rotarod test: 24 wks = controls 430,4 ± 45.14, AR113Q male mice 196.9 ± 36.78, p < 0.05 vs controls). Since changes in the muscle mass may affect the behavioural test results, both the grip strength and the rotarod tests were also statistically evaluated using body weight as covariate; however, the significant variations were similar to those presented above (not showed) indicating that the body weight had no influence on the behavioural assays performed. Interestingly, in AR113Q male mice insoluble forms of mutant ARpolyQ were only detectable in the quadriceps muscle and not in the spinal cord (Fig. 1(f)).

These results indicate that AR113Q mice develop progressive motor dysfunction and have reduced muscle strength.

### Disease-associated alteration of muscular markers in symptomatic AR113Q male mice

**Figure 2:**
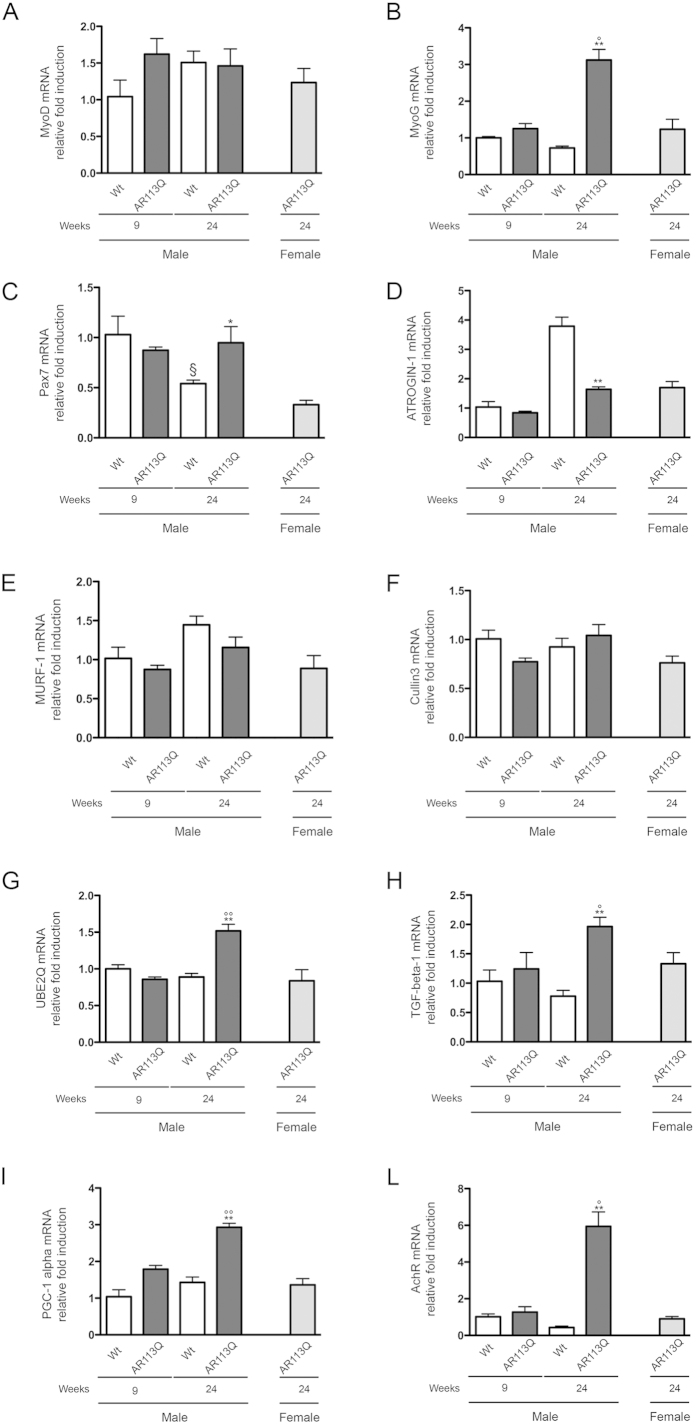
Expression of muscular markers in quadricep muscles of SBMA mouse model. RT-qPCRs were performed on total RNA extracted from quadricep muscles of male non-transgenic (Wt) mice, and of AR113Q mice at 9 (corresponding to presymptomatic stage) or 24 (corresponding to symptomatic stage) weeks of age. As additional control, quadriceps of female AR113Q mice at 24 weeks of age were used (n = 5). All animals were age-matched. Data have been normalized to the amount of GAPDH mRNA, expressed relative to the levels determined in Wt mice at 9 weeks taken as internal reference, and expressed as fold changes. Data are means ± SD of three independent replicates for Wt and AR113Q mice at 9 weeks, and of five independent replicates for Wt and AR113Q mice at 24 weeks. (**A**) RT-qPCR on MyoD mRNA expression levels in quadricep muscles. (**B**) RT-qPCR on MyoG mRNA expression levels in quadricep muscles. **p < 0.001 *vs*. 24 weeks Wt mice; °p < 0.05 *vs*. 9 weeks AR113Q mice. (**C**) RT-qPCR on Pax7 mRNA expression levels in quadricep muscles. ^§^p < 0.05 *vs*. 9 weeks Wt mice; *p < 0.05 *vs*. 24 weeks Wt mice. (**D**) RT-qPCR on Atrogin-1 mRNA expression levels in quadricep muscles. **p < 0.001 *vs*. 24 weeks Wt mice. (**E**) RT-qPCR on MURF-1 mRNA expression levels in quadricep muscles. (**F**) RT-qPCR on Cullin3 mRNA expression levels in quadricep muscles. (**G**) RT-qPCR on UBE-2Q mRNA expression levels in quadricep muscles. **p < 0.001 *vs*. 24 weeks Wt mice; °°p < 0.001 *vs*. 9 weeks AR113Q mice. (**H**) RT-qPCR on TGFβ1 mRNA expression levels in quadricep muscles. **p < 0.001 *vs*. 24 weeks Wt mice; °p < 0.05 *vs*. 9 weeks AR113Q mice. (**I**) RT-qPCR on PGC-1α mRNA expression levels in quadricep muscles. **p < 0.001 *vs*. 24 weeks Wt mice; °°p < 0.001 *vs*. 9 weeks AR113Q mice. (**L**) RT-qPCR on AchR mRNA expression levels in quadricep muscles. **p < 0.001 *vs*. 24 weeks Wt mice; °p < 0.05 *vs*. 9 weeks AR113Q mice.

In order to elucidate which changes in muscle dynamics occur in pre-symptomatic (9 wks) and symptomatic (24 wks) AR113Q mice, we analyzed the expression of proliferation/differentiation and/or atrophy markers in these animals. As an additional control, we also compared the results obtained in symptomatic AR113Q male mice with those obtained in age-matched AR113Q females. Females do not develop the pathology because they have lower levels of androgens in the serum compared to male mice^10^,^32^,^59^,^61^. The results of this first set of analyses are shown in Fig. 2. No changes in MyoD expression were observed in muscle at all ages considered (Fig. 2(a)). Conversely, MyoG (myogenin) expression was significantly increased up to three-fold in symptomatic (24 wks) AR113Q male mice over controls (e.g.: Wt mice at all ages, AR113Q male mice at presymptomatic stage 9 wks, and AR113Q female mice at 24 wks) (Fig. 2(b). We next analysed the expression of Pax-7 (a regulator of myogenesis acting on the proliferation of muscle precursors) and we found that the expression of this gene tend to decrease with age in control mice but in AR113Q male mice at 24 wks the Pax-7 mRNA were comparable to that of younger control mice (Fig. 2(c)) thus suggesting that in symptomatic animals muscle responds with an attempt to maintain elevated the myogenic pathway.

Given that AR113Q male mice show signs of muscle atrophy at 24 wks of age and are considered as symptomatic, we analysed whether this correlates with increased protein turnover mediated by the ubiquitin proteasome system (UPS). In particular, we analysed atrogin-1 and muscle RING-finger protein-1 (MuRF-1), which are two E3 ubiquitin ligases, with important regulatory functions on ubiquitin-mediated protein degradation in skeletal muscle. In addition, these two E3 ubiquitin ligases play a central role in controlling muscle size^62^. In the case of atrogin-1 expression, which in muscle it is known to increase with age^63^, we found an unexpected significant down-regulation in symptomatic (24 wks) AR113Q male mice, compared to age-matched controls (Wt male mice at 24 wks) (Fig. 2(d)). These data suggest defects in the activation of the UPS in AR113Q mice, even though MuRF-1 expression remained unchanged (Fig. 2(e)). According to this gene expression analysis UPS seems to be not massively up-regulated in these mice, as already postulated in previous reports^10^. We also analysed the expression of a third E3 ubiquitin ligase, the cullin-RING ubiquitin ligase (Cullin3), whose function is relevant for cellular stress response in a number of diseases, including neurodegenerative diseases^64^ and dominant distal myopathy^65^. However, no changes of Cullin3 expression were detected in AR113Q mice (Fig. 2(f)). Therefore, despite the fact that at 24 wks of age AR113Q male mice already show symptoms of the disease accompanied by signs of muscle atrophy and MyoG activation, the catabolic activity of muscle proteins mediated by atrogin-1, MuRF-1 and Cullin-3 ubiquitin ligases did not take place yet. On the other hand, in symptomatic (24 wks) AR113Q male mice, we surprisingly found that the expression of E2-ubiquitin ligase, the Ubiquitin-Conjugating Enzyme E2Q Family Member 1 (UBE2Q1) (Fig. 2(g)), was up-regulate.

Next, we analysed the expression of TGFβ_1_, a marker for muscle fiber damage or atrophy. We found that the expression of TGFβ_1_ is specifically upregulated only in the symptomatic (24 wks) AR113Q male mice (Fig. 2(h)), paralleling the expected deleterious effects of mutant ARpolyQ on muscle fibers of these mice. This is in line with the observation that, in a tet-repressible muscle-specific TGFβ_1_ transgenic mouse, the induction of TGFβ_1_ overexpression resulted in muscle weakness and atrophy, with the appearance of endomysial fibrosis and smaller myofibers^66^, suggesting the existence of a primary effect of TGFβ_1_ on muscle atrophy.

In addition, we observed a robust increase of PGC-1*α* mRNA expression specifically in the symptomatic (24 wks) AR113Q male mice (Fig. 2(i)). PGC-1*α* is a transcriptional co-activator (i.e. of PPAR*γ*) and regulates energy metabolism and mitochondrial biogenesis and functions. PGC-1α has been already implicated in muscle dysfunction in amyotrophic lateral sclerosis (ALS)^67^,^68^. Finally, by analysing the level of the denervation marker AchR, we found a dramatic increase of AchR gene transcription in the symptomatic (24 wks) AR113Q male mice compared to all controls mice tested as comparison (Fig. 2(l)). AchR and MyoG genes are both induced after denervation^32^. Notably, their up-regulation occurs in the absence of motoneuron loss (See Fig. 1). Collectively, these data show that, in AR113Q mice markers of muscle damage are upregulated in symptomatic animals.

### Correlation between symptoms and AR expression in muscle and in spinal cord of AR113Q male mice

**Figure 3:**
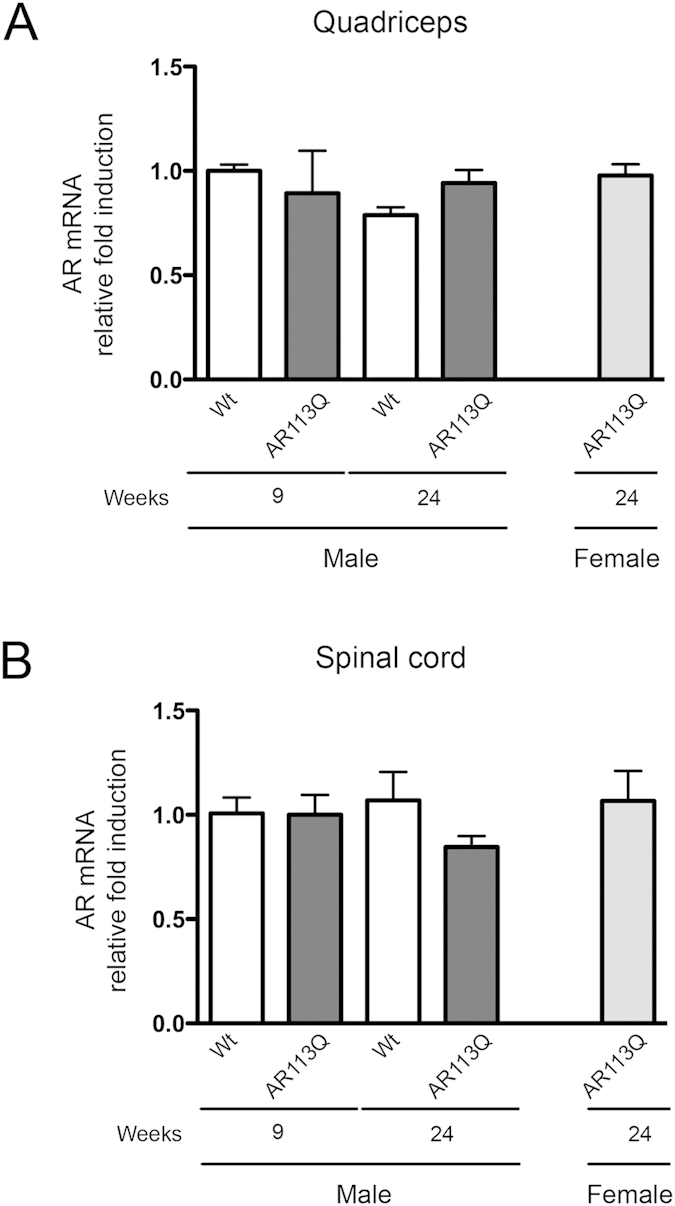
Expression of AR in quadriceps muscle and spinal cords of SBMA mouse model. RT-qPCRs were performed on total RNA extracted from quadricep muscles (**A**) or whole spinal cords (**B**) of male non-transgenic (Wt) mice, and of AR113Q mice at 9 (corresponding to presymptomatic stage) or 24 (corresponding to symptomatic stage) weeks of age. As additional control, quadriceps muscles of female AR113Q mice at 24 weeks were used (n = 5). Data have been normalized to the amount of GAPDH mRNA, expressed relative to the levels determined in Wt mice at 9 weeks taken as internal reference, and expressed as fold changes. Data are means ± SD of three independent replicates for Wt and AR113Q mice at 9 weeks, and of five independent replicates for Wt and AR113Q mice at 24 weeks. (**A**) RT-qPCR on AR mRNA expression levels in quadricep muscles. (**B**) RT-qPCR on AR mRNA expression levels in spinal cords.

Next, we asked whether ARpolyQ induces, during disease progression, a negative selection of muscle cells poorly expressing the toxic protein as a compensatory mechanism linked to detrimental effects of ARpolyQ (Fig. 1). To test our hypothesis, we analysed the levels of AR mRNA transcript and protein in skeletal muscle. The data on the analysis of AR expression are shown in Fig. 3(a). As it clearly appears in the graphs, AR expression is equivalent in all samples considered, including those derived from AR113Q male mice at 24 wks. The lack of variation in AR expression in symptomatic AR113Q male mice indicates that apparently no negative selective pressure is induced by ARpolyQ toxicity in muscle cells.

From the anatomopathological and morphological analysis reported in Fig. 1, no motoneuronal loss is present in the anterior horn of the spinal cord of AR113Q male mice at 24 wks of age. However, since symptom manifestations could also be due to its neurotoxicity at motoneuronal levels, we also decided to analyse the expression of ARpolyQ in the spinal cord of the same mice, to include molecular evidence that AR expression remains unchanged in this structure. In fact, AR expression (together with the androgen-activator machinery) in the spinal cord is mainly confined to the anterior horn motoneurons^28^,^69^,^70^ (affected in SBMA patients), while interneurons and glial cells express very low levels of AR^71^,^72^,^73^. Thus, it is conceivable that AR mRNA variations in spinal cord correlate with the motoneuronal cell modification, and are representative of motoneuron loss. The data reported in Fig. 3(b) show a mild, but not significant decrease in AR expression in AR113Q male mice at 24 wks. From these data we conclude that expression of AR in AR113Q is similar to that of control mice both in muscle and spinal cord.

### Modification of autophagic markers in muscle of symptomatic AR113Q male mice

**Figure 4:**
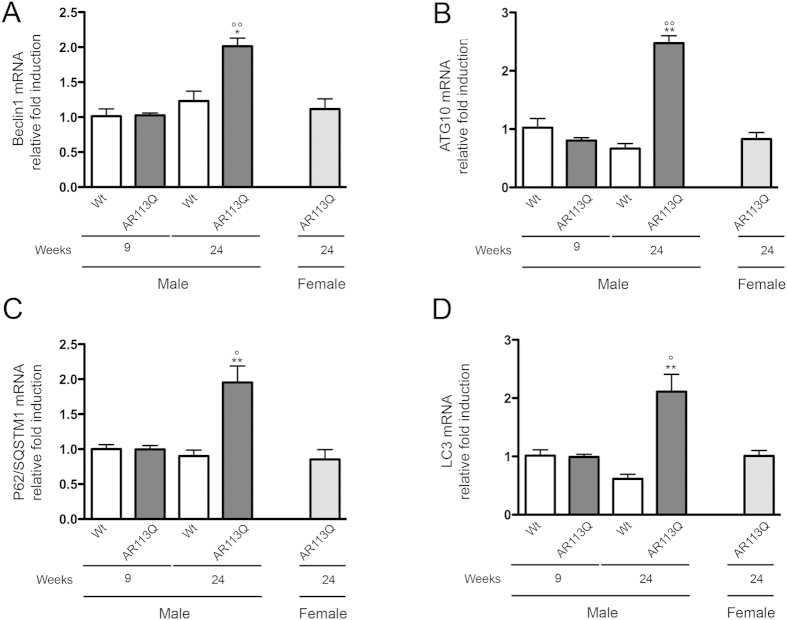
Expression of autophagic markers in quadriceps muscle of SBMA mouse model. RT-qPCRs were performed on total RNA extracted from quadricep muscles of male non-transgenic (Wt) mice, and of AR113Q mice at 9 (corresponding to presymptomatic stage) or 24 (corresponding to symptomatic stage) weeks of age. As additional control, quadriceps of female AR113Q mice at 24 weeks were used (n = 5). All animals were age-matched. Data have been normalized to the amount of GAPDH mRNA, expressed relative to the levels determined in Wt mice at 9 weeks taken as internal reference, and expressed as fold changes. Data are means ± SD of three independent replicates for Wt and AR113Q mice at 9 weeks, and of five independent replicates for Wt and AR113Q mice at 24 weeks. (**A**) RT-qPCR on Beclin1 mRNA expression levels in quadricep muscles. *p < 0.05 *vs*. 24 weeks Wt mice; °°p < 0.001 *vs*. 9 weeks AR113Q mice. (**B**) RT-qPCR on ATG10 mRNA expression levels in quadricep muscles. **p < 0.001 *vs*. 24 weeks Wt mice; °°p < 0.001 *vs*. 9 weeks AR113Q mice. (**C**) RT-qPCR on P62/SQSTM1 mRNA expression levels in quadricep muscles . **p < 0.001 *vs*. 24 weeks Wt mice; °p < 0.05 *vs*. 9 weeks AR113Q mice. (**D**) RT-qPCR on LC3 mRNA expression levels in quadricep muscles. **p < 0.001 *vs*. 24 weeks Wt mice; °p < 0.05 *vs*. 9 weeks AR113Q mice.

To better understand whether symptom onset correlates with an activation of gene expression of proteins involved in PQC system in muscle cells, we measured changes in the expression of autophagic markers in the skeletal muscle of symptomatic AR113Q male mice. It has already been reported that the autophagy master regulator gene product TFEB is up-regulated in these mice when symptoms appear^10^. TFEB increase is paralleled by the increase of some specific TFEB target genes^10^. Here, we analysed the expression of four genes encoding proteins which are essential for autophagy initiation and are critical markers for the autophagic process. Two of these genes are critical for autophagosome assembly: Beclin-1 (the ATG6 ortholog, which regulates and associates to Vps34 to induce autophagy) and ATG10 (an E2-like enzyme involved in LC3 activation and modification essential for autophagosome formation); the other two, are TFEB regulated and considered typical autophagy markers: p62/SQSTM1 (which recognizes ubiquitinated protein for their insertion into autophagosomes), and LC3 (which after its lipidation to LC3-II associates to the autophagosome membrane)^43^,^44^. We found that all autophagic markers analysed were robustly and specifically induced in skeletal muscle of symptomatic AR113Q male mice (Beclin-1 in Fig. 4(a); ATG10 in Fig. 4(b); p62/SQSTM1 in Fig. 4(c); LC3 in Fig. 4(d)) over controls; no changes were present by comparing muscles of all other mice groups included in the study.

### Induction of pro-autophagic chaperones in muscle of symptomatic AR113Q male mice

**Figure 5:**
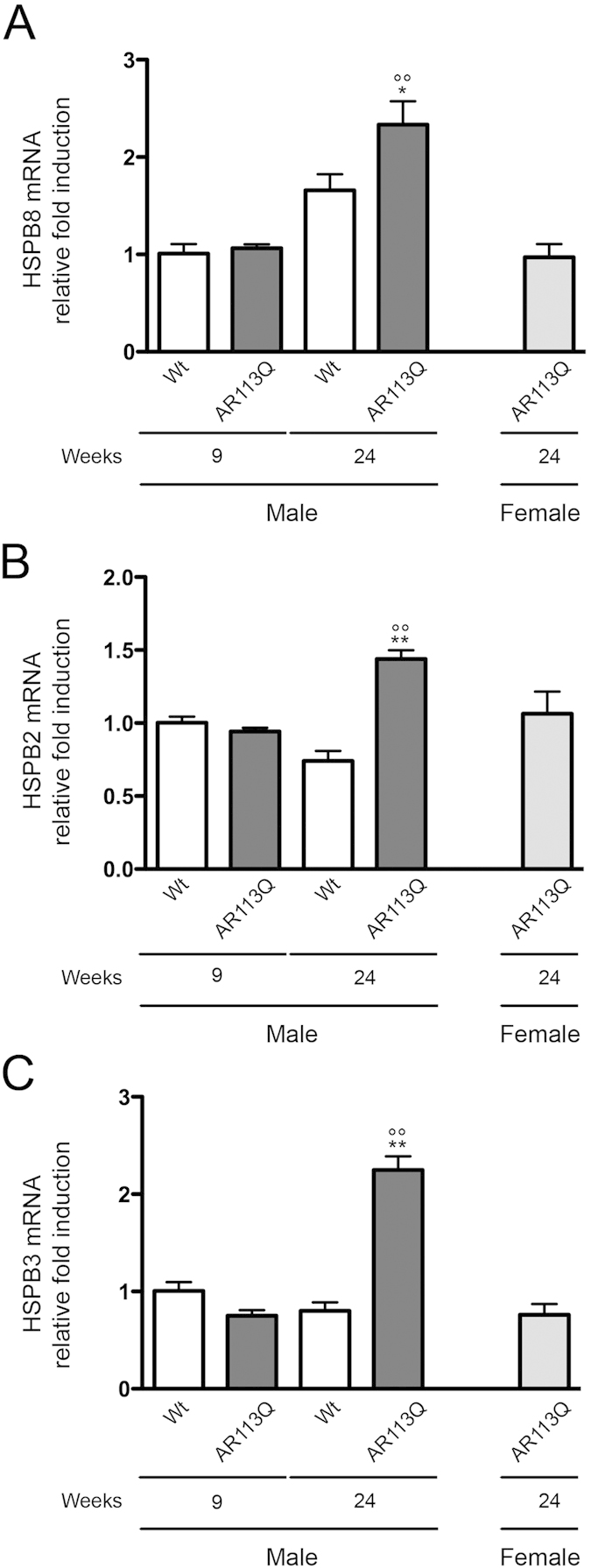
Expression of pro-autophagic chaperones in quadriceps muscle of SBMA mouse model. RT-qPCRs were performed on total RNA extracted from quadricep muscles of male non-transgenic (Wt) mice, and of AR113Q mice at 9 (corresponding to presymptomatic stage) or 24 (corresponding to symptomatic stage) weeks of age. As additional control, quadriceps of female AR113Q mice at 24 weeks were used (n = 5). All animals were age-matched. Data have been normalized to the amount of GAPDH mRNA, expressed relative to the levels determined in Wt mice at 9 weeks taken as internal reference, and expressed as fold changes. Data are means ± SD of three independent replicates for Wt and AR113Q mice at 9 weeks, and of five independent replicates for Wt and AR113Q mice at 24 weeks. (**A**) RT-qPCR on HSPB8 mRNA expression levels in quadricep muscles. *p < 0.05 *vs*. 24 weeks Wt mice; °°p < 0.001 *vs*. 9 weeks AR113Q mice. (**B**) RT-qPCR on HSPB2 mRNA expression levels in quadricep muscles. **p < 0.001 *vs*. 24 weeks Wt mice; °°p < 0.001 *vs*. 9 weeks AR113Q mice. (**C**) RT-qPCR on HSPB3 mRNA expression levels in quadricep muscles. **p < 0.001 *vs*. 24 weeks Wt mice; °°p < 0.001 *vs*. 9 weeks AR113Q mice.

We already demonstrated that one of the key protein involved in the autophagic removal of misfolded proteins (such as ARpolyQ associated to SBMA or mutant SOD1 and TDP-43 associated to ALS, etc) is the HSPB8. Expression of HSPB8 is robustly increased by UPS blockage to facilitate autophagy^53^,^54^. HSPB8 expression is also enhanced when PQC is altered in ALS especially in motoneuron and muscle cells^57^,^58^. Therefore, we investigated whether, also in the case of SBMA, HSPB8 may be involved in the response to protein toxicity in muscle. When we analysed HSPB8 expression in SBMA mice (Fig. 5(a)) we found that this gene was transcriptionally up-regulated specifically in symptomatic AR113Q male mice, while it remained unchanged in all other mice tested, and at all time points considered. HSPB8 interacts with another member of its same family, namely the chaperone HSPB2. Interestingly, HSPB2 usually conjugates with a third member of the family, the chaperone HSPB3, found mutated in an axonal type of motor neuropathy^74^; HSPB2 and HSPB3 are capable to form hetero-oligomers in a 3:1 subunit ratio^75^ and are both highly expressed in skeletal muscle where they play relevant roles during myogenic differentiation^76^,^77^. Thus, we analysed whether the induction of HSPB8 is paralleled by the induction of these other two HSPBs in response to ARpolyQ muscle toxicity. Interestingly, both genes were significantly upregulated in the muscle of symptomatic AR113Q male mice, respect all the other mice considered (Fig. 5(b,c)). Notably, these chaperones were selectively upregulated in male, but not female AR113Q mice, indicating that their upregulation mirrors androgen-dependent effects of ARpolyQ expression.

**Figure 6:**
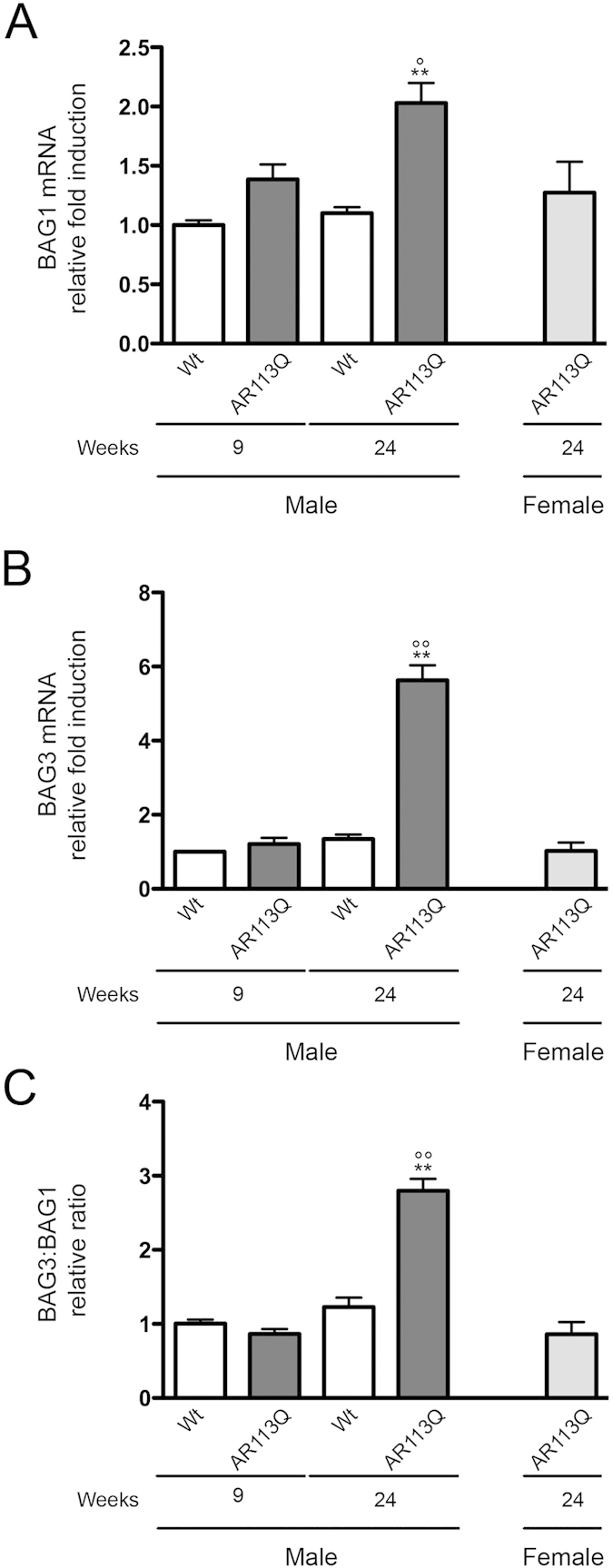
Expression of BAG1 and BAG3 co-chaperones in quadriceps muscle of SBMA mouse model. RT-qPCRs were performed on total RNA extracted from quadricep muscles of male non-transgenic (Wt) mice, and of AR113Q mice at 9 (corresponding to presymptomatic stage) or 24 (corresponding to symptomatic stage) weeks of age. As additional control, quadriceps of female AR113Q mice at 24 weeks were used (n = 5). All animals were age-matched. Data have been normalized to the amount of GAPDH mRNA, expressed relative to the levels determined in Wt mice at 9 weeks taken as internal reference, and expressed as fold changes. Data are means ± SD of three independent replicates for Wt and AR113Q mice at 9 weeks, and of five independent replicates for Wt and AR113Q mice at 24 weeks. (**A**) RT-qPCR on BAG1 mRNA expression levels in quadricep muscles. **p < 0.001 *vs*. 24 weeks Wt mice; °p < 0.05 *vs*. 9 weeks AR113Q mice. (**B**) RT-qPCR on BAG3 mRNA expression levels in quadricep muscles. **p < 0.001 *vs*. 24 weeks Wt mice; °°p < 0.001 *vs*. 9 weeks AR113Q mice. (**C**) BAG3:BAG1 relative ratio of mRNA expression levels in quadricep muscles. Data represent variations of the relative levels of BAG3 and BAG1 normalized over the relative BAG3 and BAG1 levels of age-matched 9 weeks Wt mice. **p < 0.001 *vs*. 24 weeks Wt mice; °°p < 0.001 *vs*. 9 weeks AR113Q mice.

To exert its action, HSPB8 must associate to its co-chaperone BAG3. Once misfolded species are recognized by HSPB8-BAG3, they interact with HSC70-CHIP and the misfolded substrates become ubiquitinated for p62 recognition and autophagosome insertion^49^,^53^,^55^. HSPB8 is highly unstable in the absence of BAG3. In addition, when low level of BAG3 are present, and the other co-chaperon BAG1 is induced, BAG1 preferentially associates to HSC70-CHIP to re-route protein degradation via the UPS, rather than autophagy^42^,^49^,^78^,^79^. The ratio BAG3:BAG1 is thus essential to select which PQC degradative pathway is preferred to clear misfolded proteins in a given cell type. BAG1 and BAG3 expression was then analysed in muscles of Wt mice and SBMA mice at all ages considered. We found that both co-chaperones were induced in the presence of mutant ARpolyQ in muscle of symptomatic (24 wks) AR113Q male mice, but not in all other controls tested (Fig. 6(a): BAG1 in, Fig. 6(b): BAG3). However the ratio measured was dramatically increased in favor of BAG3 (Fig. 6(c): BAG3:BAG1 ratio higher than 3) in symptomatic AR113Q male mice, while it remained similar to Wt mice in all other controls tested. Therefore, in symptomatic muscles, the autophagic pathway is preferentially activated in response to mutant ARpolyQ protein toxicity.

### Modification of proteins regulating autophagy and HSPB8-mediated response in skeletal muscle of symptomatic AR113Q male mice

**Figure 7:**
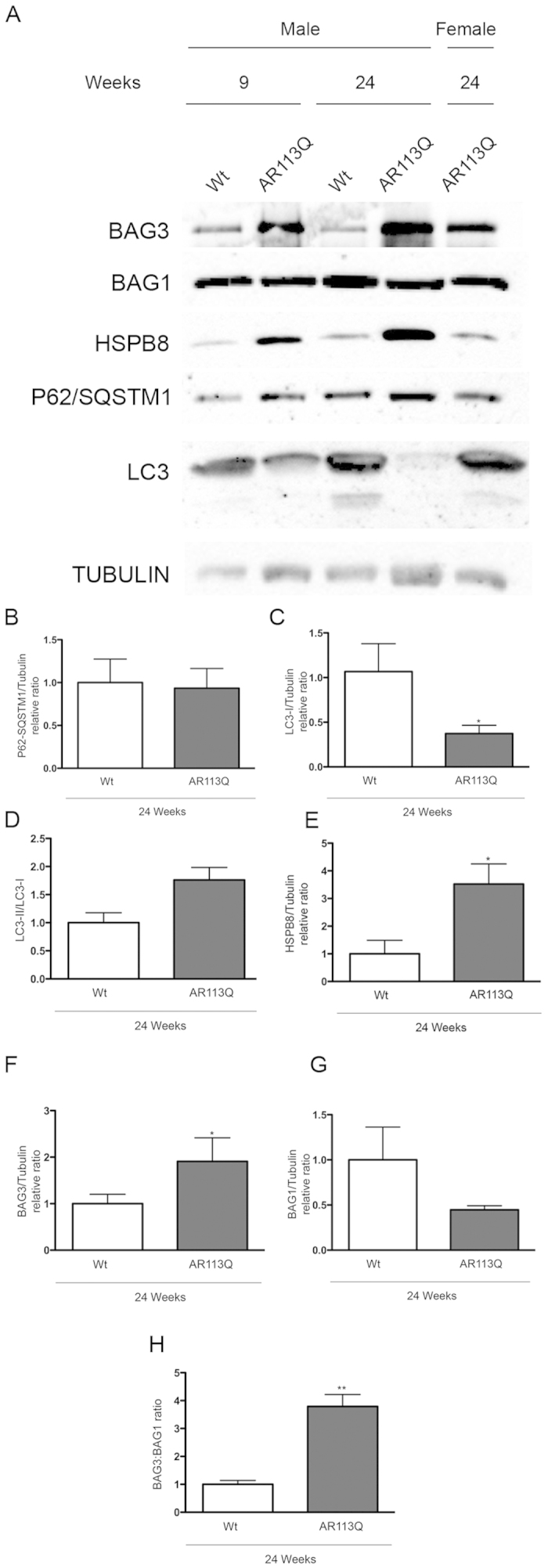
Evaluation of the levels of proteins regulating autophagy and HSPB8-mediated response in skeletal muscle of symptomatic AR113Q male mice. (**A**) Western blot analysis perfomed on quadriceps of male wt and AR113Q mice at 9 and 24 weeks. As additional control, quadriceps of female AR113Q mice at 24 weeks were used. (**B**) Quantification of p62/SQSTM1 protein expression levels in in wt (n = 3) and AR113Q (n = 3) male mice at 24 weeks. The p62/SQSTM1 protein levels were normalized using tubulin as control. (**C**) Quantification of LC3-I protein expression levels in in wt (n = 3) and AR113Q (n = 3) male mice at 24 weeks. The LC3-I protein levels were normalized using tubulin as control. *p < 0.05 *vs*. 24 weeks Wt mice. (**D**) Quantification of the LC3-II/LC3-I ratio in wt (n = 3) and AR113Q (n = 3) male mice at 24 weeks. (**E**) Quantification of HSPB8 protein expression levels in in wt (n = 3) and AR113Q (n = 3) male mice at 24 weeks. The HSPB8 protein levels were normalized using tubulin as control; *p < 0.05 *vs*. 24 weeks Wt mice. (**F**) Quantification of BAG3 protein expression levels in in wt (n = 3) and AR113Q (n = 3) male mice at 24 weeks. The BAG3 protein levels were normalized using tubulin as control; *p < 0.05 *vs*. 24 weeks Wt mice. (**G**) Quantification of BAG1 protein expression levels in in wt (n = 3) and AR113Q (n = 3) male mice at 24 weeks. The BAG1 protein levels were normalized using tubulin as control. (**H**) Quantification of the ratio between BAG3 and BAG1 protein levels in wt (n = 3) and AR113Q (n = 3) male mice at 24 weeks; **p < 0.001 *vs*. 24 weeks Wt mice.

We next evaluated whether the observed changes in gene expression of transcripts involved in regulating autophagy and HSPB8-mediated response are also paralleled by variations at protein levels. Western blot analyses (Fig. 7(a)) showed that despite the increased expression (see Fig. 4(c)), the overall protein levels of p62/SQSTM1 remained unchanged in symptomatic AR113Q male mice as compared to control wt mice of the same age (quantification in Fig. 7(b)). This is not surprising since once autophagy is activated by the expression of TFEB regulated genes (including p62/SQSTM1), the p62/SQSTM1 protein recognizes ubiquitinated substrates for their engulfment into autophagosomes, and p62 itself is processed by autophagy. Thus, p62/SQSTM1 steady state levels depend upon its transcriptional induction (during autophagy activation) and its turnover (during autophagy progression)^43^,^44^,^53^. The fact that p62/SQSTM1 does not accumulate into p62/SQSTM1 bodies suggests that the overall autophagic flux is not blocked at this initial stage by the presence of the mutant ARpolyQ in skeletal muscle. This finds further support in the observation that also LC3 expression (see Fig. 4(d), along with Beclin-1 (Fig. 4(a) and ATG10 (Fig. 4(b)) is robustly induced, proving that autophagy is activated in skeletal muscle of symptomatic AR113Q male mice. However, the overall levels of the total LC3-I are greatly reduced (Fig. 7(c)) while the LC3-II/LC3-I ratio (Fig. 7(d)) are significantly increased in skeletal muscle of symptomatic AR113Q male mice. Notably, also LC3 (after its lipidation to LC3-II) is inserted into autophagosomes to be cleared (with p62/SQSTM1 and the ubiquitinated substrated to be degraded) when autophagic flux is sustained into cells^43^,^44^,^53^. Thus, these data clearly indicate not only that autophagy is highly activated (as evidenced by increased expression of the autophagy markers reported in Fig. 4), but it is not stalled (as evidenced by reduced p62/SQSTM1 and LC3 protein levels and increased LC3-II/LC3-I ratio) in skeletal muscle of symptomatic AR113Q male mice. The proper activity of the autophagic flux may be maintained by the increased expression of the entire HSPB8-related machinery (which includes HSPB8 itself (Fig. 5(a)), BAG3 (Fig. 6(b)), and possibly the other small HSPs, HSPB2 (Fig. 5(b)) and HSPB3 (Fig. 5(c)) and also by the HSPB8 protein levels (Fig. 7(e)) in skeletal muscle of symptomatic AR113Q male mice as compared to wt male mice. Increased HSPB8 levels are accompanied by the increased levels of its co-chaperone BAG3 (Fig. 7(f)), which is absolutely required to stabilize HSPB8 protein^43^,^49^,^53^,^54^, and the increased BAG3:BAG1 ratio (Fig. 7(g,h)) which is indicative of the misfolded substrate re-routing from the UPS to autophagy^53^,^57^,^58^.

### The autophagic response in spinal cord of AR113Q male mice

**Figure 8:**
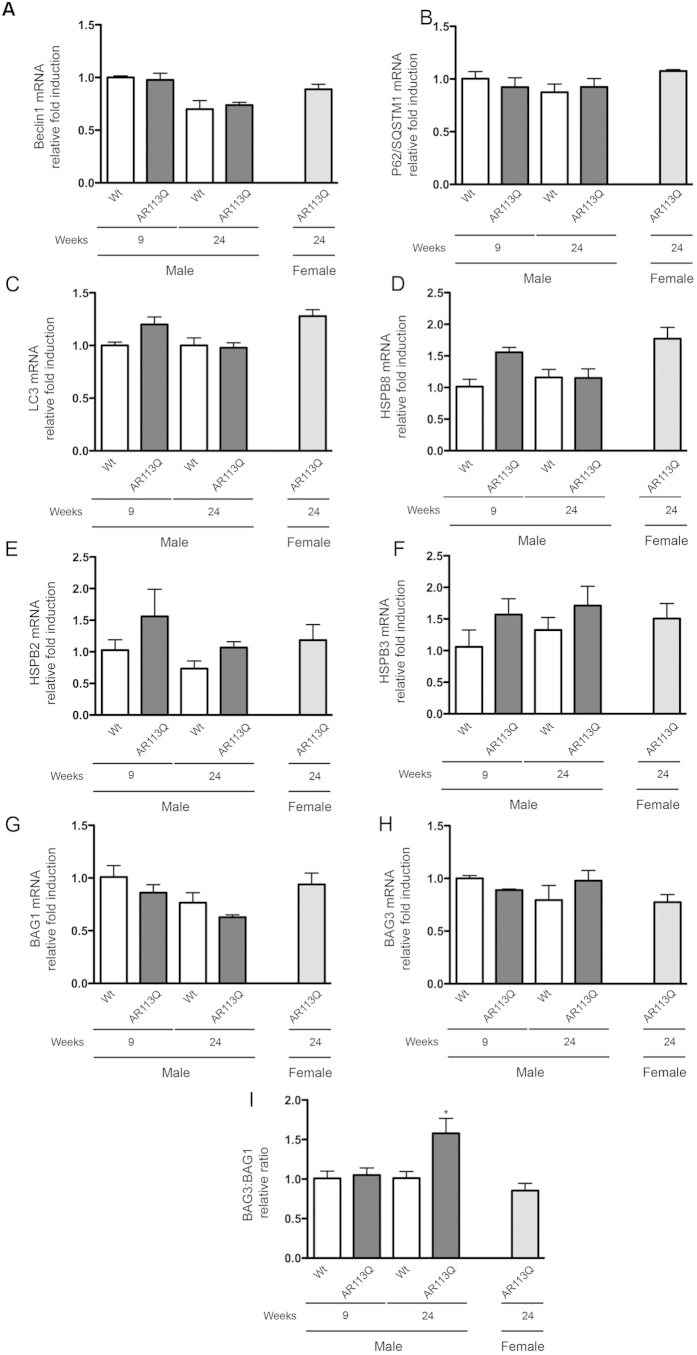
Expression of autophagic markers, chaperones, and co-chaperones in spinal cords of SBMA mouse model. RT-qPCRs were performed on total RNA extracted from whole spinal cord of male non-transgenic (Wt) mice, and of AR113Q mice at 9 weeks (corresponding to presymptomatic stage) or 24 weeks (corresponding to symptomatic stage). As additional control, spinal cord of female AR113Q mice at 24 weeks were used (n = 5). All animals were age-matched. Data have been normalized to the amount of GAPDH mRNA, expressed relative to the levels determined in Wt mice at 9 weeks taken as internal reference, and expressed as fold changes. Data are means ± SD of three independent replicates for Wt and AR113Q mice at 9 weeks, and of five independent replicates for Wt and AR113Q mice at 24 weeks. (**A**) RT-qPCR on Beclin1 mRNA expression levels in spinal cord. (**B**) RT-qPCR on P62/SQSTM1 mRNA expression levels in spinal cord. (**C**) RT-qPCR on LC3 mRNA expression levels in spinal cord. (**D**) RT-qPCR on HSPB8 mRNA expression levels in spinal cord. (**E**) RT-qPCR on HSPB2 mRNA expression levels in spinal cord. (**F**) RT-qPCR on HSPB3 mRNA expression levels in spinal cord. (**G**) RT-qPCR on BAG1 mRNA expression levels in spinal cord. (**H**) RT-qPCR on BAG3 mRNA expression levels in spinal cord. (**I**) BAG3:BAG1 relative ratio of mRNA expression levels in whole spinal cord. Data represent variations of the relative levels of BAG3 and BAG1 normalized over the relative BAG3 and BAG1 levels of age-matched 9 weeks Wt mice. *p < 0.05 *vs*. 24 weeks Wt mice.

We then evaluated whether changes in PQC markers are also present in the spinal cords of the AR113Q mice at the pre-symptomatic and symptomatic stages. When we analysed the expression of Beclin-1, p62/SQSTM1, LC3, HSPB8, HSPB2, HSPB3, BAG1 and BAG3 (Fig. 8(a–h), respectively) we failed to detect variation in the different groups tested, including symptomatic (24 wks) AR113Q male mice. The only variation observed was found in the relative BAG3:BAG1 ratio (Fig. 8(i)), which in symptomatic AR113Q male mice was in favor of the BAG3 expression suggesting the existence of a preferential activation of the autophagic pathway over the UPS pathway also in the spinal cord.

## Discussion

In the present study, we have analysed the role of the PQC system response, mediated by the HSPB8 machinery and alternative pathways in the skeletal muscle and spinal cord of mice expressing physiological levels of mutant ARpolyQ. All the gene expression analyses on the symptomatic AR113Q male mice have been performed in comparison to multiple controls, which included normal male mice and pre-symptomatic AR113Q male mice, as well as age-matched non-symptomatic AR113Q female mice, which are non-symptomatic.

Using histological analysis and behavioral test, we demonstrated that AR113Q show symptoms by 24 wks of age. At this age the AR113Q male mice showed signs of muscle atrophy, but no motoneuronal loss in the anterior horn spinal cord was detected, as demonstrated by the histopathological analysis performed on spinal cord sections. These data are in agreement with previous reports^10^,^59^,^61^.

We analyzed several markers of muscle atrophy. We found that MyoG (or myogenin) expression was greatly up-regulated in symptomatic AR113Q male mice, while the expression of Pax-7 was found to be decreased over time in wt mice, but was maintained at elevated levels also at older age in symptomatic AR113Q mice. Interestingly, elevated Pax-7 expression is indicative of an attempt to regenerate after muscle injury. Together with the observation of elevated levels of AchR, these data confirmed alteration at muscular levels and possibly an association to denervation (even if not accompained by motoneuronal loss) in the skeletal muscle of AR113Q mice^9^,^30^,^32^,^80^.

Instead, no changes were observed in the expression of the differentiation marker MyoD (as expected^80^) or the E3 ubiquitin ligases MuRF-1 and Cullin3 in the muscle of AR113Q and control mice at all ages tested. Unexpectedly, Atrogin-1 was found significantly down-regulated in symptomatic (24 wks) AR113Q male mice, possibly in response to an already activated atrophic process. It remains to be elucidated whether this atrophic process is mediated by unknown pathways involving the E2-ubiquitin ligase UBE2Q1, which is dramatically up-regulated in the muscle of symptomatic AR113Q male mice.

We then wondered whether symptomatic AR113Q male mice were characterized by modification of selected markers that we found altered in tg ALS mouse models, such as TGFβ_1_ (which correlates with muscle fiber damage or atrophy)^34^,^81^, or PGC-1*α*^67^ (which regulates energy metabolism and mitochondria biogenesis and functions). Notably, both genes were dramatically up-regulated specifically in affected mice. This finding suggests a primary role of TGF-β1 on muscle atrophy. In fact, when TGF-β1 is overexpressed it induces endomysial fibrosis and promotes myofibers atrophy^66^ accompanied by the ability of muscle cell to maintain a correct metabolism under these conditions and an attempt to overload the system by enhancing the PGC-1*α*/PPAR*γ* pathway. A role for TGF-β1 in SBMA pathology has also been proposed by other Authors demonstrating that, in the spinal cord of male transgenic mice carrying the mutant human AR, a reduced expression of the type II receptor for TGF-β, and a hampered nuclear translocation of pSmad2/3 proteins are present^82^. The blockage of the TGF-β signaling could be responsible for the motoneuron damage in SBMA since this growth factor is known to have a crucial role in the survival and function of adult neurons^83^. Despite these relevant variations in muscle in response to mutant ARpolyQ in symptomatic AR113Q male mice, we did not observe changes in the expression levels of the AR mRNA, strongly suggesting the absence of either positive (linked to trophic androgens/AR effects) or negative (linked to cytotoxic androgens/AR effects) selection of muscle cell at this relatively early stages of disease.

We then analysed whether in our colony of AR113Q mice alterations of the autophagic pathways were detectable at pre-symptomatic or symptomatic stages in male mice. It has already been reported that the autophagy master regulator TFEB is upregulated in muscles of SBMA mice and patients^10^. Here we found a mild, but not significant, up-regulation of TFEB in the symptomatic AR113Q male mice as compared to the control used (data not shown). Despite this, we found that the gene expression of all other autophagic markers analysed (Beclin-1, ATG10, p62/SQSTM1, LC3) was dramatically increased in the muscle of symptomatic AR113Q male mice. Interestingly, with regards to p62/SQSTM1 and LC3 autophagic markers, Chua and coll^10^ reported that the insoluble (but not the soluble) fraction of the p62/SQSTM1 protein and of the LC3 autophagosome-anchored lipidated form (LC3-II) were greatly enhanced in their symptomatic AR113Q male mice. In our model, we also found not only an increased p62/SQSTM1 and LC3 mRNAs expression, but a stabilization (for p62/SQSTM1) or a reduction (for LC3) in the overall levels of the corresponding coded proteins. Interestingly, both p62/SQSTM1 and LC3 proteins are normally processed by autophagy when autophagic flux is properly activated and it is not blocked. Notably, the increased turnover of total LC3 protein was accompanied by an increased LC3-II/LC3-I ratio. Thus, in agreement with a previous report^10^, collectively, these data are strongly suggestive of autophagy activation sustained by a regular flux in atrophic skeletal muscle of symptomatic AR113Q male mice.

On these bases, we demonstrated that the HSPB8-mediated PQC machinery is also greatly activated in the muscle of symptomatic AR113Q male mice. In fact, the levels of both the mRNA and protein levels of HSPB8 and its co-chaperones BAG3 were greatly up-regulated in skeletal muscle of symptomatic AR113Q male mice. Thus, the entire HSPB8-mediated autophagic machinery seems to be stimulated by the presence of mutant ARpolyQ in the skeletal muscle of SBMA affected mice. These data are consistent with those we reported in muscle of an SBMA-related motoneuron disease, the SOD1-linked familial ALS^57^,^58^. Not only HSPB8 and its co-chaperone BAG3, but also the levels of other specific HSPB8 interactors, the muscle specific small heat shock protein HSPB2 and HSPB3^75^ were found to be up-regulated in the same animals. All these modifications were accompanied by an increased BAG3:BAG1 ratio (both at mRNA and protein levels) suggestive of a preferential routing of misfolded proteins to autophagy rather than to UPS^49^,^50^, which is known to be a late event in SBMA^84^. We already demonstrated that the HSPB8-mediated PQC machinery, by facilitating the autophagic process, facilitate the clearance of different motoneuron-associated misfolded proteins (ARpolyQ, mutant SOD1, N-terminal fragment of TDP43)^43^,^44^,^53^. Altogether, the modification of the expression of specific genes encoding for proteins involved in the HSPB8-mediated PQC response here reported, may provide muscle specific biomarkers which could allow to follow the progression of disease in SBMA mice and patients during the validation of therapeutic trials.

When we analysed the spinal cord of the same animals, we did not detect variation in the expression levels of all genes included in this study, with the exception of the relative BAG3:BAG1 ratio, which was found to be increased in symptomatic AR113Q male mice, as compared to all controls tested. This finding suggests preferential activation of the autophagic pathways, instead of the UPS pathway, in the spinal cord of affected mice.

However, differently from the neuropathological features found in ALS, in which several spinal cord cell types are sites of misfolded protein toxicity (e.g.: glial cells, microglia), in SBMA the only cell types affected by the ARpolyQ neurotoxicity are the anterior horn motoneurons, which express very high levels of AR. While this allowed us to estimate that no loss of motoneurons occurs at the symptomatic stages in the mice studied here (see above), the extremely low number of motoneuron present in the spinal cord in relation to all other cell types (interneurons, glial cells microglial cells, etc.) may apparently mask a variation in gene expression. Thus, motoneurons might not be highly affected in these mice at this stage of disease, or even mild variations of expression might have been “diluted” by the large fraction of AR-negative cells (interneurons, glial cells, etc.), thus a population of unresponsive cells to ARpolyQ potential toxicity and in this AR113Q mice line, deficits to motor neurons are not observed until much later.

Therefore, all data here reported proved that the presence of mutant ARpolyQ in muscle cells induces a potent autophagic response, possibly in the attempt to clear cells from toxic misfolded species generated by the mutant protein. HSPB8-mediated PQC machinery seems to be profoundly involved in this response, in agreement with its pro-autophagic activity. These data also confirm the importance of the skeletal muscle in pathological events that are at the basis of SBMA onset and progression^16^,^32^,^33^,^85^ and support the idea that therapeutical intervention focused on the enhancement/restoration of a correct PQC activity in skeletal muscle might be beneficial in SBMA. In addition, the identification of specific autophagy related molecular markers of skeletal muscle degeneration in SBMA might represent a valuable diagnostic tool for monitoring the disease progression. In this study, we have provided an extensive characterization of the autophagic activation as well as of the HSPB8 machinery in the PQC system response in SBMA muscle.

Further research is needed to establish how muscle responds to ARpolyQ toxicity and the mechanisms capable to mediate autophagy and/or restore a normal autophagic flux in motoneurons or muscle cells may help to design novel and more selective approaches to treat SBMA patients.

## Materials and Methods

### Generation and maintenance of AR113Q knock-in mice

Animal care and experimental procedures were conducted in accordance with the Italian Institute of Technology and the University of Trento ethical committees and were approved by the Italian Ministry of Health. Generation and genotyping of mice containing androgen receptor with 113 CAG repeats in exon 1 was described previously^59^,^80^. Mice genetic background was C57Bl/6J. Females carrying one copy of AR113Q in the X chromosome were crossed with C57Bl/6J mice to maintain the colony. Mice were genotyped by PCR on tail DNA, using REDExtract-N-Amp Tissue PCR kit (Sigma-Aldrich, St Louis, MO, USA) according to manufacturer’s instructions. The mice were housed in filtered cages in a temperature-controlled room with a 12-hour light/12-hour dark cycle with ad libitum access to water and food. Body weight was followed on 5 wt and 20 AR113Q male mice.

### Histological analysis on mice spinal cord and muscle sections

Deeply anesthetized mice were transcardially perfused with 4% paraformaldehyde (PFA). Lumbar spinal cords were post-fixed for 24 h in 4% PFA and stored in PBS plus sodium azide. Paraffin embedded lumbar spinal cord was serially sectioned at 20 μm sections, mounted on slides and processed for Nissl staining. Nissl staining on spinal cord was performed on 5 wt and 6 AR113Q male mice on 7 slice/mouse (see below). Motor neurons were identified as cells positive for Nissl staining, with clear nucleus and nucleolus, with a maximum diameter greater than 20 μm leaving 100 μm between slices to avoid counting the same motor neuron. The number of motoneurons/slice in the graph is the result of the average of motoneurons per slice per mouse. Counting was performed in a blinded fashion of all the seven different slices per animal and the total number of motoneurons in these slices and the average number of motoneurons per slice were considered. The mean ± s.e.m. of the number of motor neurons in the spinal cord of 5 control mice and 6 AR113Q mice was calculated.

Muscles were collected immediately after euthanasia, flash-frozen in isopentane precooled in liquid nitrogen, and stored at −80 °C until further processing. Frozen muscles were embedded in optimal cutting temperature (OCT) compound (Tissue Tek, Sakura, Torrance, CA), and cross sections (10 μm-thick) were cut with a cryostat (CM1850 UV, Leica Microsystem, Wetzlar, Germany). Cryosections of quadriceps were stained for hematoxylin and eosin (H&E). For hematoxylin and eosin staining, sections were air-dried and incubated in hematoxylin (Sigma-Aldrich) for 3 minutes, then washed in water and incubated in eosin (Roth, Karlsruhe, Germany) for 1 minute. After incubation, sections were washed in water and dehydrated rapidly in 70, 80 and 100% ethanol, and xylene. Sections were mounted with di-Nbutyle phthalate in xylene (DPX) mounting media (Sigma-Aldrich). Images were taken using a Nikon Eclipse 90i upright microscope.

### Grip strength and Rotarod analyses

The behavior analysis was performed on 5 wt and 15 AR113Q male mice. A Grip Strength Meter (Ugo Basile Instruments) was used to measure forelimb grip strength. The grip strength meter was positioned horizontally, and mice were held by the tail and lowered toward the apparatus. Mice were allowed to grasp the smooth metal triangular pull bar with their forelimbs only and then were pulled backward. The force applied to the bar at the moment the grasp was released and recorded as the peak tension. The test was repeated 5 consecutive times within the same session, and the highest value from the 5 trials was recorded as the grip strength for that animal. The test was performed weekly from 9 to 24 weeks of age 5 control mice and 15 AR113Q SBMA mice were used in this study. Statistical analysis was performed using a two-tailed paired Student’s *t*-Test using the PRISM software (GraphPad, San Diego, CA, USA).

For rotarod analysis, mice were placed on a rotating rod using the Ugo Basile Rotarod (Ugo Basile Instruments) at constant speed of 20 rpm for maximum period of 600 seconds. The training of the animals was performed one week before the analysis. Three trials were recorded and the latency to fall was recorded for each trial and the average was used for statistical tests. The test was performed weekly from 9 to 24 weeks of age. 5 control mice and 15 AR113Q mice were used in this study. Statistical analysis was performed using a two-tailed paired Student’s *t*-Test using the PRISM software (GraphPad, San Diego, CA, USA).

In order to avoid modifications induced by the body weight of the mice in the latency of staying on the grip strength meter or on the rotarod apparatus, both the grip strength and the rotarod tests were also statistically evaluated using body weight as covariate statistical analysis on these tests was also performed using the body weight as covariate.

### Filter retardation assay

Spinal cord and quadriceps tissues of 3 control mice and 3 AR113Q mice were collected after euthanasia and store at −80 °C before processing. Tissues were pulverized using pestle and mortar and proteins were extracted using RIPA buffer, containing 150 mM NaCl, 6 mM Na_2_HPO_4_, 4 mM NaH_2_PO_4_, 2 mM EDTA pH 8, 1% Na-deoxycholate, 0.5% Triton X-100, 0.1% sodium dodecyl sulfate plus protease inhibitor cocktail (Roche). Protein lysates from spinal cord were sonicated and centrifuged at 15.000 rpm for 15 min at 4 °C. Lysates from quadriceps were homogenized with stirrer (Stirrer set RZR 2052, VWR) at 600 rpm, 20 times, and flush up and down using syringe of 22G, 25G and 28 G needles and centrifuged at 15.000 rpm for 15 min at 4 °C. Protein concentration was measured using BCA method. 50 μg of total protein were diluted in 100 μl of TBS 1X. The protein solutions were loaded in the dot-blot apparatus (Bio-Rad Laboratories, Hercules, CA, USA) and pass through a 0.2 μm acetate membrane (GE Healthcare Life Sciences, Little Chalfont, UK).

The membranes were blocked for 30 min in 5% BSA or milk in TRIS-buffered saline plus 0.1% Tween-20 (TBS-T) and incubated with anti-androgen receptor antibody (H280, Santa Cruz Biotechnology, Dallas, TX, USA) for 1 h. The membranes were rinsed three times with TBS-T for 10 min, incubated with secondary antibody conjugated with alkaline phosphatase for 30 min and washed three times with TBS-T. Signals were detected using the chemiluminescence reagent ECL (GE Healthcare Life Sciences) and proteins level in each sample was evaluated using Image J 1.48 software.

### RNA and protein extraction

Frozen spinal cord and muscle were suspended in TRI-REAGENT (Sigma-Aldrich) and homogenized with Tissue-Lyser (QIAGEN, Valencia, CA, USA) and then RNA was extracted following the TRI-REAGENT Manufacturer’s protocol. After homogenization, 1-bromo-3-chloropropane was added to ensure complete dissociation of nucleoprotein complexes and then the samples were centrifuged to obtain the phase separation. The aqueous phase, containing RNA was isolated and purified. Briefly, aqueous phase was washed with 2-propanol and subsequently with ethanol both to remove the residual phenol and to precipitate RNA, respectively. The RNA pellet was dissolved in RNase-free water (30 μL). The proteins were precipitated from the organic phase with propanol and then the pellets were washed with 0.3 M guanidine hydrochloride/95% ethanol solution (3 times). After the three washes, ethanol was added and the pellets were resuspended using 1% SDS- 8M Urea in Tris HCl pH8.0 solution (100 μL).

### Real time PCR

RNA quantification was carried out by absorbance at 260 nm, using NanoDrop 2000 (ThermoFisher Scientific, Waltham, MA, USA). Total RNA (1 μg) was treated with DNAseI (Sigma-Aldrich), and reverse transcribed into cDNA using the High-Capacity cDNA Archive Kit (Life Technologies, Carlsbad, CA, USA).

Primers for real-time PCR were designed using the Primer 3 program and were synthesized by MWG Biotech (Ebersberg, Germany) with the following sequence: mouse AchR: 5’-GTGCTGGGCTCTTTCATCTC -3′ (forward), 5′-TTCTGTGCGCGTTCTCATAC -3′ (reverse); mouse AR: 5′-ATCCCAGTCCCACTTGTGTC-3’(forward), 5′-GGTCTTCTGGGGTGGAAAGT-3′(reverse); mouse Atrogin-1: 5′-GAAGAGAGCAGTATGGGGTCA -3′ (forward), 5′-CTTGAGGGGAAAGTGAGAC-3′ (reverse); mouse ATG10: 5′-TTCACAGCAGATAGGCGATG -3′ (forward), 5′-TGCAGGTCTCGTCACTTCAG-3′ (reverse); mouse ATP6V1B2: 5′-GGCCCACAGAGAATCAGGTA -3′ (forward), 5′-GAGGGTGGGATGTAGGGTTT -3′ (reverse); mouse BAG1: 5′-GAAACACCGTTGTCAGCACT -3′ (forward), 5′-GCTCCACTGTGTCACACTC-3′ (reverse); mouse BAG3: 5′-ATGGACCTGAGCGATCTCA -3′ (forward), 5′-CACGGGGATGGGGATGTA -3′ (reverse); mouse Beclin1: 5′-TGAAATCAATGCTGCCTGGG-3′ (forward), 5′-CCAGAACAGTATAACGGCAACTCC -3′ (reverse); mouse Cullin3: 5′-CACACCAAAGTGCAACATCC -3′ (forward); 5′-CCAACACCAACCTCAGATCC -3′ (reverse); mouse HSPB2: 5′-GCTCAGTGAAGGCAAGTTCC -3′ (forward), 5′-CAGGACATAGGTGCGACAGA-3′(reverse); mouse HSPB3: 5′-CATCATCATCCAGACCTTCG -3′ (forward), 5′-ACTTCCACCACCAAGATTCC-3′ (reverse); mouse HSPB8: 5′-ATACGTGGAAGTTTCAGG CA -3′ (forward), 5′-TCCTTTGACCTAACGCAACC -3′ (reverse); mouse MAP-LC3b: 5′-CGTCCTGGACAAGACCA -3′ (forward), 5′-CCATTCACCAGGAGGAA -3′ (reverse); mouse MyoD: 5′-GGCTACGACACCGCCTACTA -3′ (forward), 5′-GTGGAGATGCGCTCCACTAT -3′ (reverse); mouse Myogenin: 5′-GGGCAATGCACTGGAGTT -3′ (forward), 5′-CACGATGGACGTAAGGGAGT -3′ (reverse); mouse MURF: 5′-ACCTGCTGGTGGAAAACATC-3′(forward), 5’-AGGAGCAAGTAGGCACCTCA-3′(reverse); mouse p62/SQSTM-1: 5′-AGGGAACACAGCAAGCT -3′ (forward), 5′-GCCAAAGTGTCCATGTTTCA -3′ (reverse); mouse Pax7: 5′-GTATGGCCAAACTGCTGTTGAT -3′ (forward), 5′-GGAGTGTTCCCCAAGCTTCA-3′(reverse); mouse PGC1-α: 5′-GGAATGCACCGTAAATCTGC -3′ (forward), 5′-TTCTCAAGAGCAGCGAAAGC-3′ (reverse); mouse TGFβ1: 5′-GAAGGACCTGGGTTGGAAGT -3′ (forward), 5′-CGGGTTGTGTTGGTTGTAGA -3′ (reverse); mouse UBE2Q: 5′-GGAACAGTTGCCTGGATGTT -3′ (forward), 5′-CTGGTGGGTGTAGGCAGAAT -3′ (reverse); mouse GAPDH: 5′-CCAGAACATCATCCCTGCAT -3′ (forward), 5′-CAGTGAGCTTCCCGTTCA -3′ (reverse).

The evaluated efficiency of each set of primers was close to 100%. Real-time PCR was performed using the CFX 96 Real Time System (Bio-Rad Laboratories, Hercules, CA, USA), in a 10 *μ*L total volume, using the iTaq SYBR Green Supermix (Bio-Rad Laboratories), and with 500nmol primers. PCR cycling conditions were as follows: 94 °C for 10 min, 40 cycles at 94 °C for 15 s, and 60 °C for 1 min. Melting curve analysis was performed at the end of each PCR assay to control specificity. Data was expressed as Ct values and used for the relative quantification of targets with the ΔΔCt calculation to give N-fold changes in gene expression (2^−ΔΔCt^). Values were normalized to those of GAPDH.

### Western blot analysis

Protein concentration was determined using Pierce 660nM protein assay reagent with Ionic Detergent Compatibility Reagent (IDCR) (Thermo-Fisher Scientific). WB analysis were performed using 10 and 15% SDS-polyacrylamide gel electrophoresis.

20 μg of proteins for each sample were loaded in the gel. The gels were electrotransferred to nitrocellulose membranes using the Trans-turbo Transfer system (Bio-Rad Laboratories). The membranes were incubated with blocking solution (5% non-fat dried milk in TBS-T) for 1h, and then incubated o/n with the following primary antibodies: home-made rabbit polyclonal anti-BAG3 (kindly provided by Prof. Serena Carra, Università degli studi di Modena e Reggio Emilia, Italy; 1:6000); rabbit polyclonal anti-BAG1 (Santa Cruz, Dallas, TX, USA, 1:500); home-made rabbit polyclonal anti-HSPB8 (provided by Dr. J. Landry, Quebec, Canada; 1:1000); rabbit polyclonal anti-p62 (Abcam, Cambridge, UK; dilution 1:2000); rabbit polyclonal anti-LC3 (Sigma-Aldrich; dilution 1:1000); mouse monoclonal anti- alpha tubulin (Sigma-Aldrich; dilution 1:2000). After washing, the membranes were incubated for 1 hour with the TBS-T solution containing the peroxidase-conjugated antibody (goat anti-rabbit, goat anti-mouse Santa-Cruz, 1:5000).

The immunoreactive bands were detected with ECL-prime (GE-Healthcare, Maidstone, Uk) and the images were obtained with ChemiDoc XRS system (Bio-Rad Laboratories).

### Statistical analysis

Statistical analysis was performed using a one-tailed Student’s *t*-Test for two group comparisons and two-way ANOVA for three or more groups comparisons using the PRISM software (GraphPad, San Diego, CA, USA). Specific group pair(s) statistical difference was determined by the Bonferroni *post-hoc* test.

## Additional Information

**How to cite this article**: Rusmini, P. *et al.* Aberrant Autophagic Response in The Muscle of A Knock-in Mouse Model of Spinal and Bulbar Muscular Atrophy. *Sci. Rep.* **5**, 15174; doi: 10.1038/srep15174 (2015).
